# The role of whole brain radiation therapy in the management of melanoma brain metastases

**DOI:** 10.1186/1748-717X-9-143

**Published:** 2014-06-22

**Authors:** Michael A Dyer, Nils D Arvold, Yu-Hui Chen, Nancy E Pinnell, Timur Mitin, Eudocia Q Lee, F Stephen Hodi, Nageatte Ibrahim, Stephanie E Weiss, Paul J Kelly, Scott R Floyd, Anand Mahadevan, Brian M Alexander

**Affiliations:** 1 Harvard Radiation Oncology Program, Boston, MA, USA; 2 Department of Radiation Oncology, Dana-Farber/Brigham & Women’s Cancer Center, Boston, MA, USA; 3 Harvard Medical School, Boston, MA, USA; 4 Department of Biostatistics and Computational Biology, Dana-Farber Cancer Institute, Boston, MA, USA; 5 Department of Radiation Oncology, Massachusetts General Hospital, Boston, MA, USA; 6 Center for Neuro-Oncology, Dana-Farber/Brigham & Women’s Cancer Center, Boston, MA, USA; 7 Melanoma Disease Center, Department of Medical Oncology, Dana-Farber Cancer Institute, Boston, MA, USA; 8 Fox Chase Cancer Center, Philadelphia, PA, USA; 9 Temple University School of Medicine, Philadelphia, PA, USA; 10 Radiotherapy Department, Cork University Hospital, Cork, Ireland; 11 University College Cork, Cork, Ireland; 12 Department of Radiation Oncology, Beth Israel Deaconess Medical Center, Boston, MA, USA

**Keywords:** Melanoma, Brain metastases, Radiosurgery, Whole brain radiation therapy

## Abstract

**Background:**

Brain metastases are common in patients with melanoma, and optimal management is not well defined. As melanoma has traditionally been thought of as “radioresistant,” the role of whole brain radiation therapy (WBRT) in particular is unclear. We conducted this retrospective study to identify prognostic factors for patients treated with stereotactic radiosurgery (SRS) for melanoma brain metastases and to investigate the role of additional up-front treatment with whole brain radiation therapy (WBRT).

**Methods:**

We reviewed records of 147 patients who received SRS as part of initial management of their melanoma brain metastases from January 2000 through June 2010. Overall survival (OS) and time to distant intracranial progression were calculated using the Kaplan-Meier method. Prognostic factors were evaluated using the Cox proportional hazards model.

**Results:**

WBRT was employed with SRS in 27% of patients and as salvage in an additional 22%. Age at SRS > 60 years (hazard ratio [HR] 0.64, p = 0.05), multiple brain metastases (HR 1.90, p = 0.008), and omission of up-front WBRT (HR 2.24, p = 0.005) were associated with distant intracranial progression on multivariate analysis. Extensive extracranial metastases (HR 1.86, p = 0.0006), Karnofsky Performance Status (KPS) ≤ 80% (HR 1.58, p = 0.01), and multiple brain metastases (HR 1.40, p = 0.06) were associated with worse OS on univariate analysis. Extensive extracranial metastases (HR 1.78, p = 0.001) and KPS (HR 1.52, p = 0.02) remained significantly associated with OS on multivariate analysis. In patients with absent or stable extracranial disease, multiple brain metastases were associated with worse OS (multivariate HR 5.89, p = 0.004), and there was a trend toward an association with worse OS when up-front WBRT was omitted (multivariate HR 2.56, p = 0.08).

**Conclusions:**

Multiple brain metastases and omission of up-front WBRT (particularly in combination) are associated with distant intracranial progression. Improvement in intracranial disease control may be especially important in the subset of patients with absent or stable extracranial disease, where the competing risk of death from extracranial disease is low. These results are hypothesis generating and require confirmation from ongoing randomized trials.

## Background

Metastatic tumors from primary sites outside the central nervous system (CNS) are the most frequent intracranial neoplasms. Melanoma is among the primary tumors with the highest propensity for metastasis to the brain [1]. Up to half of patients with melanoma develop brain metastases, and once brain metastases are diagnosed, median survival time is estimated to range between 3.4 and 13.2 months [2,3]. For individual patients, survival depends on a number of factors, and there has been much effort, both with brain metastases in general and with melanoma brain metastases specifically, to identify the factors that prognosticate for overall survival (OS) [4-11]. This information can be useful for patients and for their physicians who base treatment decisions in part on prognostic factor data.

Treatment options for patients with melanoma brain metastases include whole brain radiation therapy (WBRT), surgery, stereotactic radiosurgery (SRS), systemic therapy, some combination of these treatments, and supportive measures alone [3,12]. SRS has become increasingly common, even for patients with multiple brain metastases [13-22], but some authors caution against the regular omission of up-front WBRT [20,22]. Randomized data support adding WBRT to SRS for initial management of patients with brain metastases (not specific to patients with melanoma as the primary cancer) for the improvement of intracranial disease control, but not overall survival [23-25]. The translation of intracranial disease control to overall survival depends on competing risks from extracranial disease as well as the availability and efficacy of salvage options, suggesting that the impact of WBRT may differ depending on these factors. Data from a randomized trial of SRS alone versus SRS and WBRT specifically in patients with melanoma is not yet available [26], and retrospective studies are vulnerable to selection bias [13-18,27].

In this retrospective study of melanoma patients treated with SRS for brain metastases, we sought to evaluate the potential utility of WBRT, given the lack of randomized evidence specific to this subpopulation, and to identify prognostic factors associated with OS and distant intracranial progression. Though we cannot eliminate selection bias altogether in a retrospective study examining the utility of WBRT, we sought to mitigate the effects of unmeasured confounders by leveraging inter-institutional practice variation.

## Methods

### Patient population

This study was approved by the Dana-Farber/Harvard Cancer Center Institutional Review Board.

We identified 243 consecutive patients with a histological diagnosis of melanoma who were treated with SRS for brain metastases from January 2000 through June 2010 at the Dana-Farber/Brigham & Women’s Cancer Center (DF/BWCC) or the Beth Israel Deaconess Medical Center (BIDMC). One patient was excluded due to lack of follow-up data. Thirty-five patients in whom SRS was performed only on a surgical resection cavity were also excluded. An additional 65 patients were excluded because SRS was performed only as salvage therapy. As *a priori* intention-to-treat could not be assigned retrospectively, salvage SRS was defined as any SRS performed greater than 3 months from the date of first treatment for brain metastases (i.e. the date of WBRT or surgery when these treatments were used prior to SRS) or any SRS performed for progression of intracranial disease (even if within 3 months of the first treatment). We reviewed records of the remaining 147 patients who received SRS as the initial management of their melanoma brain metastases. The follow-up schedule was not uniform in all cases, but the typical approach was to obtain brain magnetic resonance imaging (MRI) 6–8 weeks after SRS alone followed by MRI every 3 months thereafter if stable and every three months after WBRT.

#### Stereotactic radiosurgical technique

At DF/BWCC, SRS was performed using the Novalis™ linear accelerator-based radiosurgery platform. Prior to 2009, all patients were immobilized with the use of a fixed head frame. In 2009, conventional frame-based radiosurgery was replaced by frameless delivery using the thermoplastic BrainLAB cranial mask immobilization system. At BIDMC, SRS was performed using the X-Knife® linear accelerator-based radiosurgery platform or, beginning in 2005, the Cyberknife® platform.

### Statistical analysis

Distant intracranial progression was defined as the presence of a new enhancing lesion consistent with a brain metastasis or leptomeningeal enhancement outside of the SRS target volume on any MRI after the date of SRS. Estimates of time to intracranial progression and OS were calculated using the Kaplan-Meier method. Time to distant intracranial progression was defined as the interval from SRS to the date of first distant intracranial progression (censored at the date of last MRI demonstrating no evidence of progression). OS was defined as the interval from SRS to the date of death (censored at the date of last clinical follow-up). The effects of clinical and demographic covariates on intracranial progression and OS were estimated using a Cox proportional hazards model. Variables with a p-value < 0.1 on univariate analysis were used to construct a multivariate model. (Age, as a continuous variable, was included in the multivariate model for OS regardless of significance on univariate analysis, given that for people in general, age is the most significant prognosticator for OS.) All other statistical tests used a significance level of 0.05 (two-sided) and a 95% confidence interval (95% CI). Analyses were performed using SAS (version 9.2) and R (version 3.0.3).

Clinical factors that were analyzed included patient age, Karnofsky Performance Status (KPS), number of brain metastases (one versus multiple), time from initial diagnosis of melanoma to SRS, use of up-front WBRT, the *status* of extracranial disease (absent or stable versus progressive), and the *extent* of extracranial metastases (limited versus extensive). In terms of extracranial disease *status*, “progressive” was defined as any evidence of new or progressing systemic disease on restaging computed tomography (CT) scan (by review of radiology reports and physician notes) within the 3 months prior to SRS. If no CT was available in the 3 months prior to SRS, then any restaging CT performed in the month following SRS was used. To define the *extent* of extracranial metastases, each patient was assigned a number between 0 and 6 based on evidence of any current (i.e. at the time of SRS) or past melanoma metastases to the following 6 sites: liver, lung, adrenal glands, other visceral organs, bone, or other distant site (e.g. lymph nodes or subcutaneous tissue). For example, if a particular patient had metastases to lungs, liver, and distant subcutaneous tissue, then the patient would be assigned a “3”. The median number of extracranial body sites affected by metastatic disease was used to dichotomize the *extent*-of-extracranial-metastases variable into “limited extracranial metastases” (less than or equal to the median number of body sites affected by metastatic melanoma) and “extensive extracranial metastases” (greater than the median number of body sites affected by metastatic melanoma).

## Results

### Patient characteristics

The median follow-up time for survivors was 23 months. The frequency of clinical covariates is shown in Table 1. Median age was 60 years. Eighty patients (54%) had KPS of 90% or 100%; 61 patients (41%) had KPS of 70% or 80%; and 6 patients (4%) had KPS of 50% or 60%. Thirty-five patients (24%) had absent or stable extracranial metastases, while 112 patients (76%) had progressive extracranial disease. The median number of extracranial sites affected by metastatic melanoma was 2. Eighty-six patients (59%) had “limited extracranial metastases” (i.e. ≤ 2 sites affected by metastatic melanoma), and 61 patients (41%) had “extensive extracranial metastases” (i.e. ≥ 3 extracranial sites affected by metastatic melanoma) At DF/BWCC, 54% of patients were initially treated with WBRT in addition to SRS (i.e. “up-front WBRT”), while only 3% of patients had up-front WBRT at BIDMC. An additional 13% of patients at DF/BWCC and 30% of patients at BIDMC received salvage WBRT. Only 2 of 59 patients (3%) with one brain metastasis received up-front WBRT, while 37 of 88 patients (42%) with multiple brain metastases did so. The use of up-front WBRT was associated with treatment center (Fisher’s exact test: p < 0.0001) and multiple brain metastases (p < 0.0001). The median number of brain metastases for patients receiving WBRT up front was 4 (IQR 3–5) while those treated with SRS alone was 1 (IQR 1–2).

**Table 1:** Patient characteristics and clinical covariates

			**Median (IQR)**	**Range**
Age at stereotactic radiosurgery (years)			60 (51 – 68)	28 – 92
Time from primary Melanoma to Brain Metastasis (months)			39 (17 – 69)	0 – 347
Diameter of largest brain Metasasis (mm)			11 (8 – 21)	3 – 55
			**n (%)**	
**Gender:**				
	Male		99 (67%)	
	Female		48 (33%)	
Extent of extracranial metastases:*				
	0		12 (8%)	
	1	Limited	28 (19%)	86 (59%)
	2		46 (31%)	
	3		35 (24%)	
	4	Extensive	18 (12%)	61 (41%)
	5		7 (5%)	
	6		1 (1%)	
**Extracranial disease status:**				
	Absent		12 (8%)	
	Present and stable		23 (16%)	
	Present and progressive		112 (76%)	
**Number of brain metastases:**				
	1		59 (40%)	
	2 – 3		53 (36%)	
	> 3 (4–10)		35 (24%)	
**Karnofsky performance status:**				
	50 – 60		6 (4%)	
	70 – 80		61 (41%)	
	90 – 100		80 (54%)	
**Initial treatment:**				
			*DF/BWCC*	*BIDMC*
	SRS Alone		29 (43%)	66 (84%)
	SRS + WBRT		23 (34%)	0 (0%)
	SRS + Surgery		2 (3%)	11 (14%)
	SRS + Surgery + WBRT		14 (21%)	2 (3%)
**Whole-brain radiation therapy:**				
			*DF/BWCC*	*BIDMC*
	Up-Front (with SRS)		37 (54%)	2 (3%)
	As Salvage Therapy		9 (13%)	24 (30%)
	Never		22 (32%)	53 (67%)
**Systemic treatment with SRS:**^**†**^				
	None		78 (53%)	
	Temozolomide		41 (28%)	
	Other		28 (19%)	
**Melanoma-GPA score:**				
	0 – 1		23 (16%)	
	2		41 (28%)	
	3		44 (30%)	
	4		39 (27%)	

*Abbreviations:IQR* interquartile range, *DF/BWCC* Dana-Farber/Brigham & Women’s Cancer Center, *BIDMC* Beth Israel Deaconess Medical Center, *SRS* stereotactic radiosurgery, *WBRT* whole brain radiation therapy, Melanoma-GPA melanoma-specific Graded Prognostic Assessment for brain metastases.

*To define the “*extent* of extracranial metastases”, each patient was assigned a number between 0 and 6 based on evidence of melanoma metastases to the following 6 sites: liver, lung, adrenal glands, other visceral organs, bone, or other distant site (e.g. lymph nodes or subcutaneous tissue). The median number of extracranial body sites affected by metastatic melanoma is 2. “Limited extracranial metastases” was defined as ≤ 2 extracranial body sites affected by metastatic melanoma. “Extensive extracranial metastases” was defined as ≥ 3 extracranial body sites affected by metastatic melanoma.

^†^Systemic Treatment with SRS = systemic treatment given after SRS and before disease progression.

### Intracranial progression

**Figure 1:**
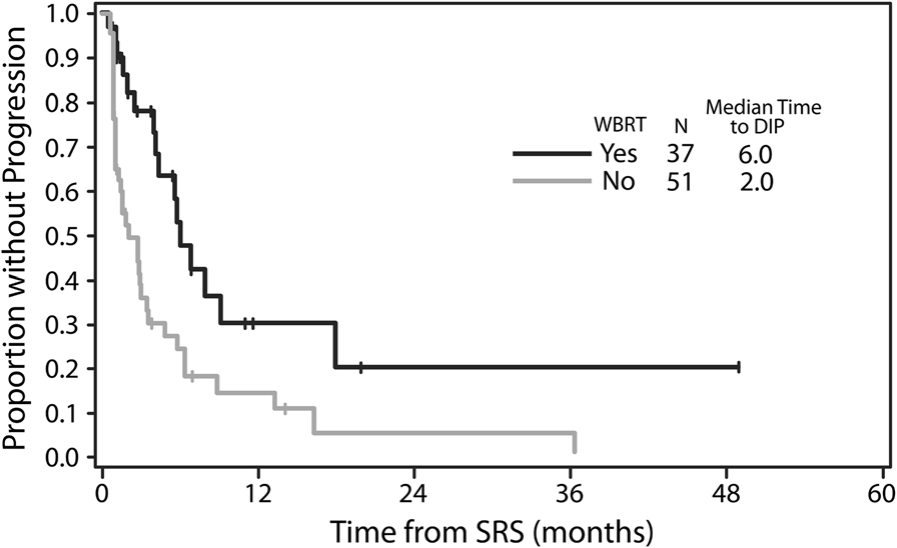
**Kaplan-Meier curves for distant intracranial progression by use of WBRT.** (Only the 88 patients with greater than 1 brain metastasis are included in the figure). (Logrank: p = 0.003). *Abbreviations:* WBRT = whole brain radiation therapy; SRS = stereotactic radiosurgery.

Fifty-six patients had distant failure prior to any local failure (i.e. progression within the SRS treatment field); 20 had distant and local failure at the same time; and 27 people had local failure first (10 of these 27 people went on to develop distant intracranial progression at a later time). Therefore, distant intracranial progression occurred in a total of 86 patients (59%). Median time to distant intracranial progression was 4.3 months. Table 2 lists the results of Cox regression analysis for time to distant intracranial progression. On multivariate analysis, age > 60 years (hazard ratio [HR] 0.64, p = 0.05), multiple brain metastases (HR 1.90, p = 0.008), and omission of up-front WBRT (HR 2.24, p = 0.005) were associated with distant intracranial progression. In patients with multiple brain metastasis, median time to distant intracranial progression was 2.0 months in patients in whom WBRT was initially omitted (i.e. those treated with SRS alone or SRS and surgery as initial treatment), and 6.0 months in patients treated with up-front WBRT (in addition to up-front SRS [and, in some cases, up-front surgery as well]) (p = 0.003). In patients with a single brain metastasis, however, the median time to distant intracranial progression was approximately 5 months, both in patients treated with up-front WBRT and in patients in whom WBRT was initially omitted. Figure 1 shows the Kaplan-Meier curves for distant intracranial progression by use of up-front WBRT in the subset of 88 patients with multiple brain metastases. In terms of local failure (i.e. growth of the lesion[s] within the SRS treatment field), on Cox univariate analysis, both WBRT (HR 2.56, p = 0.02) and diameter of largest brain metastasis (diameter >1 cm: HR 2.70, p = 0.03) were associated with local failure first (i.e. local progression taking place prior to distant intracranial progression), but WBRT was not significant on multivariate analysis or when number of brain metastases was added to the model. Of the patients who did not receive up-front WBRT and who subsequently failed, 42% received WBRT as salvage therapy.

**Table 2:** Model selection for time to distant intracranial progression

	**Univariate analysis**			**Multivariate analysis**		
	**HR**	**95% CI**	**p value**	**HR**	**95% CI**	**p value**
Age at SRS >60 years	**0.66**	**0.43 – 1.01**	**0.05**	**0.64**	**0.41 – 0.99**	**0.05**
Time from primary to brain met <39 mo.	1.16	0.76 – 1.77	0.50			
Extensive ECM (vs. Limited)*	1.20	0.77 – 1.89	0.43			
Progressive ECD (vs. absent/stable)	1.15	0.70 – 1.87	0.58			
Number of brain metastases >1	**1.45**	**0.93 – 2.24**	**0.10**	**1.90**	**1.18 – 3.06**	**0.008**
Diameter of brain metastasis >1 cm	0.85	0.55 – 1.30	0.44			
KPS 50 – 80 (vs. 90 or 100)	0.90	0.58 – 1.41	0.65			
Omission of up-front WBRT	**1.54**	**0.91 – 2.60**	**0.09**	**2.24**	**1.27 – 3.94**	**0.005**

*Abbreviations:HR* hazard ratio, *CI* confidence interval, *SRS* stereotactic radiosurgery, *Met* metastasis, *ECM* extracranial metastases, *ECD* extracranial disease, *KPS* Karnofsky Performance Status, *WBRT* whole brain radiation therapy.

*See Materials and Methods section and Table 1 for definitions of “extensive” and “limited” extracranial metastases.

### Overall survival

Death occurred in 135 patients (92%), and median OS was 7.3 months. Table 3 lists the results of Cox regression analysis for OS. On univariate analysis, the factors associated with worse OS were extensive extracranial metastases (HR 1.86, p = 0.0006), multiple brain metastases (HR 1.40, p = 0.06), and KPS ≤ 80 (HR 1.58, p = 0.01). Extensive extracranial metastases and KPS remained significantly associated with OS on multivariate analysis. When either or both omission of up-front WBRT and multiple brain metastases were included in the model, neither was significant; similarly, when the OS analysis was performed in the subgroup of patients with multiple brain metastases, omission of up-front WBRT was not significant. The final multivariate model for OS in the general cohort contains three variables: age (as a continuous variable; HR 0.99, p = 0.37), extensive extracranial metastases (HR 1.78, p = 0.001), and KPS ≤ 80 (HR 1.52, p = 0.02). Median survival for patients with extensive extracranial metastases and KPS ≤ 80 was 3.8 months, compared to 10.8 months in patients with limited extracranial metastases and KPS 90 or 100.

**Table 3:** Model selection for overall survival

	**Univariate analysis**			**Multivariate analysis**		
	**HR**	**95% CI**	**p value**	**HR**	**95% CI**	**p value**
Age at SRS (in years, continuous)*	1.00	0.98 – 1.01	0.46	0.99	0.98 – 1.01	0.37
Age at SRS >60 years	0.94	0.67 – 1.32	0.71			
Time from primary to brain met <39 mo.	1.11	0.79 – 1.55	0.56			
Extensive ECM (vs. Limited)^†^	**1.86**	**1.31 – 2.64**	**0.0006**	**1.78**	**1.25 – 2.53**	**0.001**
Progressive ECD (vs. absent/stable)	1.22	0.82 – 1.82	0.32			
Number of brain metastases >1	**1.40**	**0.99 – 1.98**	**0.06**			
Diameter of brain metastasis >1 cm	1.24	0.89 – 1.75	0.21			
KPS 50 – 80 (vs. 90 or 100)	**1.58**	**1.12 – 2.21**	**0.01**	**1.52**	**1.08 – 2.15**	**0.02**
Omission of up-front WBRT	0.96	0.66 – 1.41	0.85			

*Abbreviations:HR* hazard ratio, *CI* confidence interval, *SRS* stereotactic radiosurgery, *Met* metastasis, *ECM* extracranial metastases, *ECD* extracranial disease, *KPS* Karnofsky Performance Status, *WBRT* whole brain radiation therapy.

*Age (as a continuous variable) was included in multivariate models for overall survival regardless of significance on univariate analysis.

^†^See Materials and Methods section and Table 1 for definitions of extensive and limited extracranial metastases.

#### Subset analysis in patients with absent or stable extracranial disease

**Figure 2:**
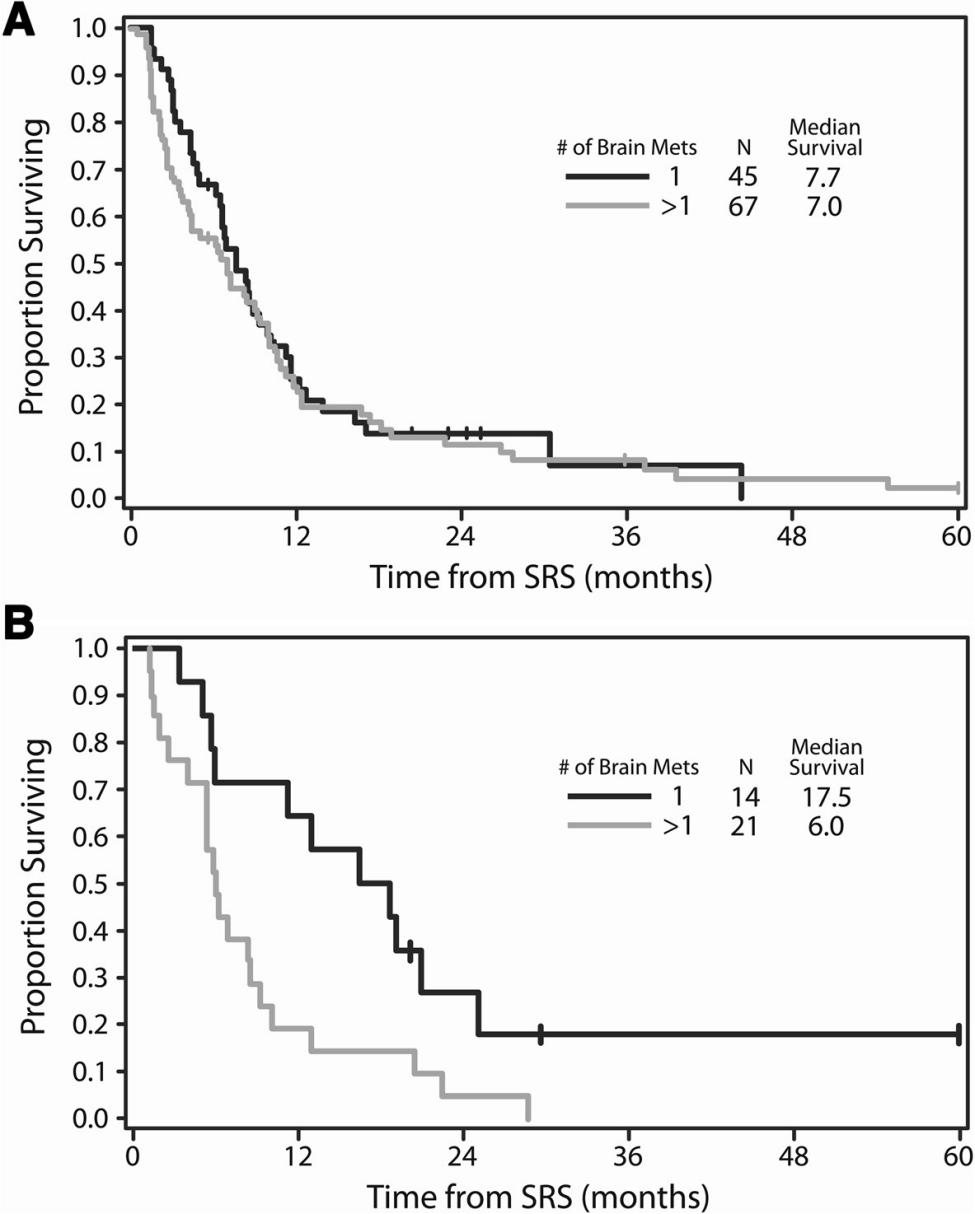
**Kaplan-Meier curves for overall survival by number of brain metastases, separated by extracranial disease status. **(**A**: Subset of patients with progressive extracranial disease. **B**: Subset of patients with absent or stable extracranial disease). (One patient with progressive extracranial disease in the multiple brain metastasis group was censored at 75 months, and one patient with absent/stable disease in the single brain metastasis group was censored at 102 months [not shown in figure]). (Logrank for 112 patients with progressive extracranial disease: p = 0.59. Logrank for 35 patients with absent/stable extracranial disease: p = 0.01). *Abbreviations:* SRS = stereotactic radiosurgery.

We repeated the Cox regression analyses in the subset of 35 patients with absent or stable extracranial disease. Multiple brain metastases (HR 4.64 [95% CI 1.54 – 13.94], p = 0.006) and omission of up-front WBRT (HR 3.14 [95% CI 1.02-9.68, p = 0.05) were associated with distant intracranial progression in the multivariate model. The final multivariate model for OS in this subset contained age (as a continous variable; HR 1.02, p = 0.24), multiple brain metastases (HR 5.89 [95% CI 1.79 – 19.43], p = 0.004), and omission of up-front WBRT (HR 2.56 [95% CI 0.91 – 7.23], p = 0.08). Therefore, there was a trend toward worse OS with omission of up-front WBRT. When any WBRT (including up-front and salvage WBRT) was put in the Cox model in place of the up-front-WBRT variable, there was no such trend. Figure 2 shows the Kaplan-Meier curves for OS by number of brain metastases in the subset of 35 patients with absent/stable extracranial disease (Figure 2B) and in the subset of 112 patients with progressive extracranial disease (Figure 2A). Given that *extent* of extracranial metastases (limited vs. extensive) – not the *status* of extracranial disease (absent/stable vs. progressive) – was associated with OS in the entire cohort, a subset analysis was also performed in patients with limited extracranial disease. When both number of brain metastases and up-front WBRT were entered into the Cox regression model for OS in the 86 patients with limited extracranial disease, there was a trend toward an association with worse OS for multiple brain metastases, but WBRT was not significant.

## Discussion

In our series of patients with melanoma brain metastases treated with SRS, multiple brain metastases and omission of up-front WBRT were associated with distant intracranial progression. Whether or not the factors associated with freedom from distant intracranial progression would also be expected to improve OS depends partly on the issue of competing risk, that is, whether intracranial disease or extracranial disease is the main driver of mortality. If mortality is driven by intracranial disease, then factors that improve intracranial disease control, such as fewer brain metastases and the initial use of WBRT with SRS, would also be expected to improve OS. On the other hand, if extracranial disease is driving mortality, then extracranial disease burden (e.g. the extent of extracranial metastases) and status (e.g. whether the extracranial disease is stable or progressing) would be expected to affect mortality, while number of brain metastases and WBRT would not. Indeed, in our study, none of the factors that improved freedom from distant intracranial progression (or freedom from local failure) was in our final model for OS. Extracranial disease, more so than intracranial disease factors or progression, appears to drive mortality in the overall cohort.

In our analysis of the subset of patients with absent or stable extracranial disease, the factors associated with improved survival were those related to intracranial disease. Specifically, of the three factors associated with distant intracranial progression in the entire cohort, one (i.e. multiple brain metastases) was also associated with poor OS in the subset analysis (p = 0.004), and another (i.e. omission of up-front WBRT) demonstrated a trend toward such an association (p = 0.08). Extracranial disease status may, therefore, modify the effect of intracranial disease burden (and control) on OS, such that the number of brain metastases (and the use of up-front WBRT) is prognostic for survival in patients with absent or stable extracranial metastases, but not in patients with progressive extracranial disease.

Recently we argued that for certain cancers, such as lung cancer, the presence versus absence of extracranial disease may be an appropriate surrogate for overall extracranial disease burden, whereas a more granular approach may be necessary for other cancers [28]. In our series of 51 patients treated with SRS for breast cancer brain metastases, extracranial disease status, measured as absent or stable versus progressive (as opposed to absent versus present), was our most robust prognostic factor for OS and added pertinent additional information to the established prognostic index, the Graded Prognostic Assessment (GPA) [28]. For patients with melanoma, where 92% and 76% of the patients in our cohort had present and progressive extracranial disease, respectively, an even more granular measure may be necessary. Indeed the “extent of extracranial metastases” variable that we defined for this study may better capture the overall burden of extracranial disease in patients with metastatic melanoma. Even this variable is an imperfect proxy for overall disease burden, however. Other groups have also shown the importance of extracranial disease as a prognostic factor for OS in patients with melanoma brain metastases, and some did so by measuring lactate dehydrogenase (LDH) level, which may also capture the extent of extracranial disease in a more granular fashion [9-11,15,17]. Adding some surrogate for extracranial disease burden to existing prognostic indices that do not already include such a factor may improve these indices.

The data regarding the use of WBRT for patients with melanoma are mixed, with some authors arguing that melanoma is a radioresistant tumor with limited benefit from non-SRS radiation treatments [14]. Some studies have shown no benefit of WBRT for melanoma brain metastases [13-15,27,29], while others suggest WBRT may improve intracranial disease control [17] or survival [18]. Several studies acknowledge that the absence of an observed effect of WBRT on intracranial disease control or survival might be due to selection bias. While we are not able to eliminate unmeasurable selection bias in this retrospective study, we hopefully diminished its effects with respect to WBRT by including patients from two treatment centers with different institutional practices. One center frequently performed both SRS and WBRT as part of initial management of melanoma brain metastases (particularly in those with more than one brain metastasis), while the other rarely did so. Even with this attempt to diminish selection bias in our study, the use of WBRT was as strongly associated with multiple brain metastases as it was with treatment center. Our results do, however, suggest that WBRT added to SRS improves freedom from distant intracranial progression (especially in patients with multiple brain metastases), providing some evidence that melanoma is not as uniformly radioresistant (and that the utility of WBRT in melanoma is not as limited) as some have argued. Whether the reduction in distant intracranial failure is clinically important in the overall course of the disease is in question, but the effect of WBRT in reducing failure is less so, given our data.

One limitation of our study is the degree to which we are able to address the question of whether improved intracranial control matters. Whether improvements in intracranial control translate to a more meaningful clinical benefit (such as overall survival) depends not only on competing risk, but also on the efficacy of salvage options. We could not directly compare up-front WBRT to salvage WBRT, as only 42% of eligible patients received salvage WBRT. Our data does not provide direct evidence to either support or refute any claim on this issue, but the trend toward better OS with the initial use of WBRT in patients with absent or stable extracranial disease may suggest imperfect salvage options. Furthermore, while our institutional practice has been to obtain surveillance MRI every 3 months, this could not be standardized in a retrospective study and the potential impacts of more frequent monitoring on salvage options and efficacy is unknown.

Multiple randomized trials of WBRT and SRS versus SRS alone in patients with brain metastases from various primary cancers (mostly lung and breast cancers) suggest that WBRT does not improve OS [23-25]. Yet in certain primary cancers, especially in those that are more likely to have progressive intracranial disease as a source of mortality, the improvements in intracranial control caused by WBRT may theoretically contribute to a survival benefit. Such cancers may act more aggressively in the CNS or have a more limited risk of extracranial disease progression. Whether melanoma fits into this category is currently unknown. Conversely, if extracranial disease progression is the primary driver of mortality, then WBRT may have a very limited role in addition to focal therapies, as prophylaxis against distant brain metastases may not be clinically relevant in such a scenario. Additionally, more frequent MRI surveillance may increase the efficacy of salvage therapy, obviating the need for upfront WBRT. A randomized, phase III trial of local therapy (neurosurgical resection and/or SRS) with or without WBRT for patients with melanoma brain metastases is currently being conducted by the Australia and New Zealand Melanoma Trials Group and the Trans Tasman Radiation Oncology Group [26]. In this trial, randomization is stratified by extracranial disease status. Such randomized trials that stratify by extracranial disease status may have the best potential for confirming our findings and the findings from other retrospective studies.

## Conclusions

In summary, we identified multiple brain metastases and the omission of up-front WBRT (particularly in those with multiple brain metastases) as factors associated with distant intracranial progression after SRS. The effect of these factors on OS was modified by extracranial disease status, such that multiple brain metastases and (to a degree that could be characterized as a trend) omission of up-front WBRT were associated with worse OS in patients with absent or stable extracranial disease, but not in those with progressive extracranial disease. The data also confirm the significance of KPS for OS and suggest that extracranial disease burden (as measured by the number of extracranial body sites affected by melanoma metastases) may be an important factor for OS in the general population of patients undergoing SRS for melanoma brain metastases. As the ability to control systemic disease in patients with metastatic melanoma improves, the contribution of intracranial disease burden and control in helping to determine OS may increase.

## Abbreviations

WBRT: Whole brain radiation therapy; SRS: Wtereotactic radiosurgery; OS: Overall survival; HR: Hazard ratio; KPS: Karnofsky performance status; CNS: Central nervous system; DF/BWCC: Dana-Farber/Brigham & Women’s Cancer Center; BIDMC: Beth Israel Deaconess Medical Center; MRI: Magnetic resonance imaging; 95% CI: 95% confidence interval; CT: Computed tomography; GPA: Graded Prognostic Assessment; LDH: Lactate dehydrogenase.

## Competing interests

Dr. Hodi reports non-paid consultancy and institute receipt of clinical trial support from Bristol-Myers Squibb, non-paid consultancy and institute receipt of clinical trial support from Merck, non-paid consultancy and institute receipt of clinical trial support from Genetech, personal fees from Amgen, and personal fees from Novartis. However, none of these companies or the relationships Dr. Hodi has with these companies will benefit or suffer from the publication of this manuscript.

## Authors’ contributions

MAD participated in study design, data entry, data analysis, and writing of the manuscript. NDA, EQL, FSH, NI, SEW, and SRF helped with study design and drafting of the manuscript. YC carried out statistical analysis and participated in study design. NEP, TM, and PJK helped with data entry/database design, study design, and drafting of the manuscript. AM and BMA oversaw the study and participated in study design and writing of the manuscript. All authors read and approved the final manuscript.

## References

[B1] Gavrilovic IT Posner JB Brain metastases: epidemiology and pathophysiology J Neurooncol 2005 75 5 14 16215811 10.1007/s11060-004-8093-6

[B2] Sperduto PW Kased N Roberge D Xu Z Shanley R Luo X Sneed PK Chao ST Weil RJ Suh J Bhatt A Jensen AW Brown PD Shih HA Kirkpatrick J Gaspar LE Fiveash JB Chiang V Knisely JP Sperduto CM Lin N Mehta M Summary report on the graded prognostic assessment: an accurate and facile diagnosis-specific tool to estimate survival for patients with brain metastases J Clin Oncol 2012 30 419 425 22203767 10.1200/JCO.2011.38.0527 PMC3269967

[B3] Carlino MS Fogarty GB Long GV Treatment of melanoma brain metastases: a new paradigm Cancer J 2012 18 208 212 22453023 10.1097/PPO.0b013e31824b2890

[B4] Gaspar L Scott C Rotman M Asbell S Phillips T Wasserman T McKenna WG Byhardt R Recursive partitioning analysis (RPA) of prognostic factors in three Radiation Therapy Oncology Group (RTOG) brain metastases trials Int J Radiat Oncol Biol Phys 1997 37 745 751 9128946 10.1016/s0360-3016(96)00619-0

[B5] Weltman E Salvajoli JV Brandt RA de Morais Hanriot R Prisco FE Cruz JC de Oliveira Borges SR Wajsbrot DB Radiosurgery for brain metastases: a score index for predicting prognosis Int J Radiat Oncol Biol Phys 2000 46 1155 1161 10725626 10.1016/s0360-3016(99)00549-0

[B6] Lorenzoni J Devriendt D Massager N David P Ruiz S Vanderlinden B Van Houtte P Brotchi J Levivier M Radiosurgery for treatment of brain metastases: estimation of patient eligibility using three stratification systems Int J Radiat Oncol Biol Phys 2004 60 218 224 15337559 10.1016/j.ijrobp.2004.02.017

[B7] Sperduto PW Berkey B Gaspar LE Mehta M Curran W A new prognostic index and comparison to three other indices for patients with brain metastases: an analysis of 1,960 patients in the RTOG database Int J Radiat Oncol Biol Phys 2008 70 510 514 17931798 10.1016/j.ijrobp.2007.06.074

[B8] Sperduto PW Chao ST Sneed PK Luo X Suh J Roberge D Bhatt A Jensen AW Brown PD Shih H Kirkpatrick J Schwer A Gaspar LE Fiveash JB Chiang V Knisely J Sperduto CM Mehta M Diagnosis-specific prognostic factors, indexes, and treatment outcomes for patients with newly diagnosed brain metastases: a multi-institutional analysis of 4,259 patients Int J Radiat Oncol Biol Phys 2010 77 655 661 19942357 10.1016/j.ijrobp.2009.08.025

[B9] Staudt M Lasithiotakis K Leiter U Meier F Eigentler T Bamberg M Tatagiba M Brossart P Garbe C Determinants of survival in patients with brain metastases from cutaneous melanoma Br J Cancer 2010 102 1213 1218 20372154 10.1038/sj.bjc.6605622 PMC2856002

[B10] Nieder C Marienhagen K Geinitz H Grosu AL Can current prognostic scores reliably guide treatment decisions in patients with brain metastases from malignant melanoma? J Cancer Res Ther 2011 7 47 51 21546742 10.4103/0973-1482.80458

[B11] Davies MA Liu P McIntyre S Kim KB Papadopoulos N Hwu WJ Hwu P Bedikian A Prognostic factors for survival in melanoma patients with brain metastases Cancer 2011 117 1687 1696 20960525 10.1002/cncr.25634

[B12] Eichler AF Loeffler JS Multidisciplinary management of brain metastases Oncologist 2007 12 884 898 17673619 10.1634/theoncologist.12-7-884

[B13] Mori Y Kondziolka D Flickinger JC Kirkwood JM Agarwala S Lunsford LD Stereotactic radiosurgery for cerebral metastatic melanoma: factors affecting local disease control and survival Int J Radiat Oncol Biol Phys 1998 42 581 589 9806518 10.1016/s0360-3016(98)00272-7

[B14] Chang EL Selek U Hassenbusch SJ 3rd Maor MH Allen PK Mahajan A Sawaya R Woo SY Outcome variation among “radioresistant” brain metastases treated with stereotactic radiosurgery Neurosurgery 2005 56 936 945 discussion 936–945 15854241

[B15] Yu C Chen JC Apuzzo ML O’Day S Giannotta SL Weber JS Petrovich Z Metastatic melanoma to the brain: prognostic factors after gamma knife radiosurgery Int J Radiat Oncol Biol Phys 2002 52 1277 1287 11955740 10.1016/s0360-3016(01)02772-9

[B16] Selek U Chang EL Hassenbusch SJ 3rd Shiu AS Lang FF Allen P Weinberg J Sawaya R Maor MH Stereotactic radiosurgical treatment in 103 patients for 153 cerebral melanoma metastases Int J Radiat Oncol Biol Phys 2004 59 1097 1106 15234044 10.1016/j.ijrobp.2003.12.037

[B17] Brown PD Brown CA Pollock BE Gorman DA Foote RL Stereotactic radiosurgery for patients with “radioresistant” brain metastases Neurosurgery 2002 51 656 665 discussion 665–657 12188943

[B18] Buchsbaum JC Suh JH Lee SY Chidel MA Greskovich JF Barnett GH Survival by radiation therapy oncology group recursive partitioning analysis class and treatment modality in patients with brain metastases from malignant melanoma: a retrospective study Cancer 2002 94 2265 2272 12001126 10.1002/cncr.10426

[B19] Liew DN Kano H Kondziolka D Mathieu D Niranjan A Flickinger JC Kirkwood JM Tarhini A Moschos S Lunsford LD Outcome predictors of Gamma Knife surgery for melanoma brain metastases. Clinical article J Neurosurg 2011 114 769 779 20524829 10.3171/2010.5.JNS1014

[B20] Cranmer LD Jeter JM Morgan SS Hersh EM Stea B Whole-brain radiation therapy in melanoma: an open question Lancet Oncol 2010 11 13 author reply 13–14 20129127 10.1016/S1470-2045(09)70346-8

[B21] Hara W Tran P Li G Su Z Puataweepong P Adler JRJ Soltys SG Chang SD Gibbs IC Cyberknife for brain metastases of malignant melanoma and renal cell carcinoma Neurosurgery 2009 64 A26 A32 19165071 10.1227/01.NEU.0000339118.55334.EA

[B22] Manon R O’Neill A Knisely J Werner-Wasik M Lazarus HM Wagner H Gilbert M Mehta M Phase II trial of radiosurgery for one to three newly diagnosed brain metastases from renal cell carcinoma, melanoma, and sarcoma: an Eastern Cooperative Oncology Group study (E 6397) J Clin Oncol 2005 23 8870 8876 16314647 10.1200/JCO.2005.01.8747

[B23] Chang EL Wefel JS Hess KR Allen PK Lang FF Kornguth DG Arbuckle RB Swint JM Shiu AS Maor MH Meyers CA Neurocognition in patients with brain metastases treated with radiosurgery or radiosurgery plus whole-brain irradiation: a randomised controlled trial Lancet Oncol 2009 10 1037 1044 19801201 10.1016/S1470-2045(09)70263-3

[B24] Kocher M Soffietti R Abacioglu U Villa S Fauchon F Baumert BG Fariselli L Tzuk-Shina T Kortmann RD Carrie C Ben Hassel M Kouri M Valeinis E van den Berge D Collette S Collette L Mueller RP Adjuvant whole-brain radiotherapy versus observation after radiosurgery or surgical resection of one to three cerebral metastases: results of the EORTC 22952–26001 study J Clin Oncol 2011 29 134 141 21041710 10.1200/JCO.2010.30.1655 PMC3058272

[B25] Aoyama H Shirato H Tago M Nakagawa K Toyoda T Hatano K Kenjyo M Oya N Hirota S Shioura H Kunieda E Inomata T Hayakawa K Katoh N Kobashi G Stereotactic radiosurgery plus whole-brain radiation therapy vs stereotactic radiosurgery alone for treatment of brain metastases: a randomized controlled trial JAMA 2006 295 2483 2491 16757720 10.1001/jama.295.21.2483

[B26] Fogarty G Morton RL Vardy J Nowak AK Mandel C Forder PM Hong A Hruby G Burmeister B Shivalingam B Dhillon H Thompson JF Whole brain radiotherapy after local treatment of brain metastases in melanoma patients–a randomised phase III trial BMC Cancer 2011 11 142 21496312 10.1186/1471-2407-11-142 PMC3107806

[B27] Samlowski WE Watson GA Wang M Rao G Klimo PJ Boucher K Shrieve DC Jensen RL Multimodality treatment of melanoma brain metastases incorporating stereotactic radiosurgery (SRS) Cancer 2007 109 1855 1862 17351953 10.1002/cncr.22605

[B28] Dyer MA Kelly PJ Chen YH Pinnell NE Claus EB Lee EQ Weiss SE Arvold ND Lin NU Alexander BM Importance of extracranial disease status and tumor subtype for patients undergoing radiosurgery for breast cancer brain metastases Int J Radiat Oncol Biol Phys 2012 83 054 10.1016/j.ijrobp.2012.01.054 22704705

[B29] Marcus DM Lowe M Khan MK Lawson DH Crocker IR Shelton JW Melton A Maynard N Delman KA Carlson GW Rizzo M Prognostic factors for overall survival after radiosurgery for brain metastases from melanoma Am J Clin Oncol 2013 E-pub ahead of print 10.1097/COC.0b013e318280d7be 23428955

